# Phlebotomine Sand Fly Fauna and *Leishmania* Infection in the Vicinity of the Serra do Cipó National Park, a Natural Brazilian Heritage Site

**DOI:** 10.1155/2015/385493

**Published:** 2015-02-22

**Authors:** Rosana Silva Lana, Érika Monteiro Michalsky, Consuelo Latorre Fortes-Dias, João Carlos França-Silva, Fabiana de Oliveira Lara-Silva, Ana Cristina Vianna Mariano da Rocha Lima, Daniel Moreira de Avelar, Juliana Cristina Dias Martins, Edelberto Santos Dias

**Affiliations:** ^1^Laboratório de Leishmanioses, Centro de Pesquisas René Rachou, Fundação Oswaldo Cruz, Avenida Augusto de Lima 1715, 30190-002 Belo Horizonte, MG, Brazil; ^2^Diretoria de Pesquisa e Desenvolvimento, Fundação Ezequiel Dias, 30510-010 Belo Horizonte, MG, Brazil; ^3^Departamento de Parasitologia, ICB, Universidade Federal Minas Gerais, 31270-901 Belo Horizonte, MG, Brazil; ^4^Secretaria Municipal de Saúde, 35830-000 Jaboticatubas, MG, Brazil

## Abstract

In the New World, the leishmaniases are primarily transmitted to humans through the bites of *Leishmania*-infected *Lutzomyia* (Diptera: Psychodidae) phlebotomine sand flies. Any or both of two basic clinical forms of these diseases are endemic to several cities in Brazil—the American cutaneous leishmaniasis (ACL) and the American visceral leishmaniasis (AVL). The present study was conducted in the urban area of a small-sized Brazilian municipality (Jaboticatubas), in which three cases of AVL and nine of ACL have been reported in the last five years. Jaboticatubas is an important tourism hub, as it includes a major part of the Serra do Cipó National Park. Currently, no local data is available on the entomological fauna or circulating *Leishmania*. During the one-year period of this study, we captured 3,104 phlebotomine sand flies belonging to sixteen *Lutzomyia* species. In addition to identifying incriminated or suspected vectors of ACL with DNA of the etiological agent of AVL and vice versa, we also detected *Leishmania* DNA in unexpected *Lutzomyia* species. The expressive presence of vectors and natural *Leishmania* infection indicates favorable conditions for the spreading of leishmaniases in the vicinity of the Serra do Cipó National Park.

## 1. Introduction

Leishmaniases are a complex of parasitic diseases caused by flagellated protozoa belonging to genus* Leishmania* Ross, 1903. About 310 million individuals are at risk of contracting any of the various clinical forms of leishmaniasis, and some 2 million new cases occur yearly [1]. In the New World, the leishmaniases are primarily transmitted to humans through the bites of* Lutzomyia* (Diptera: Psychodidae) phlebotomine sand flies [2]. Two basic clinical forms of leishmaniases are known—the American cutaneous leishmaniasis (ACL) and the American visceral leishmaniasis (AVL). Each form has different* Leishmania* species as etiological agents and distinct* Lutzomyia* species as transmitting vectors. Only the phlebotomine females play a role in the infection process due to their bloodsucking feeding habits.

*Leishmania* (*Viannia*)* braziliensis*,* Le.* (*V.*)* guyanensis*, and* Le.* (*Leishmania*)* amazonensis* are the most important causative agents of ACL [3]. A number of phlebotomine sand fly species have been incriminated as ACL vectors, including* Lutzomyia (Nyssomyia) intermedia* (Lutz & Neiva, 1912),* Lu. migonei* (França, 1920),* Lu. (N.) whitmani* (Antunes & Coutinho, 1939),* Lu. (Pintomyia) fisheri* (Pinto, 1926),* Lu. (P.) pessoai* (Coutinho & Barreto, 1940), and* Lu. (N.) flaviscutellata* (Mangabeira, 1942) [4].

*Leishmania (Leishmania) infantum* (syn.* Le. chagasi*) is recognized as etiological agent of AVL [5]. In Brazil, two* Lutzomyia* species are involved in AVL transmission—*Lutzomyia longipalpis* (Lutz & Neivai, 1912) as the primary and* Lu. cruzi* (Mangabeira, 1938) as the secondary vector [6].

Between 2009 and 2013, about 113,600 and 18,000 new cases of ACL and AVL, respectively, were compulsorily reported to the Brazilian Ministry of Health [7]. In large- and medium-sized cities, public health services have been put in place, providing the necessary logistic support to epidemiological studies and control actions. In smaller towns, however, this support is incipient.

The present study was developed in Jaboticatubas, a small-sized municipality and an important tourism hub located in the Brazilian state of Minas Gerais. The municipality includes about 65% of the area of the Serra do Cipó National Park, an important natural Brazilian heritage site. This park is known for its rich and diversified flora and fauna, tracking trails, waterfalls, and archaeological sites with cave paintings. As it attracts hundreds of visitors every year, it is an important economic asset. In the 2009–2013 period, three human cases of AVL and nine cases of ACL were reported in Jaboticatubas [7]. Presently, the transmission potential in the region is unknown. Therefore, the aim of this work was to survey the local presence of possible phlebotomine sand fly vectors and of etiological agents of leishmaniases. To our knowledge, this is the first entomological study developed in Jaboticatubas and in Serra do Cipó National Park surroundings.

## 2. Material and Methods

### 2.1. Study Area

Jaboticatubas (19°30′49′′S, 43°44′42′′W) is located at the* Serra do Espinhaço*, in Southeastern Brazil. The municipality extends across a 1,114.1 km^2^ area, at an average altitude of 772 m. According to the latest survey, it had a population of 17,134 inhabitants, which is equivalent to 15.4 inhabitants/km^2^ [8]. The climate is tropical, characterized by cool summers and a well-defined dry season. This mountainous region is rich in quartzite rocks and outcrops of limestone, with predominance of sandy soil with rock fields, covered by riparian forest and savannah vegetation [9].

### 2.2. Entomological Survey

**Figure 1:**
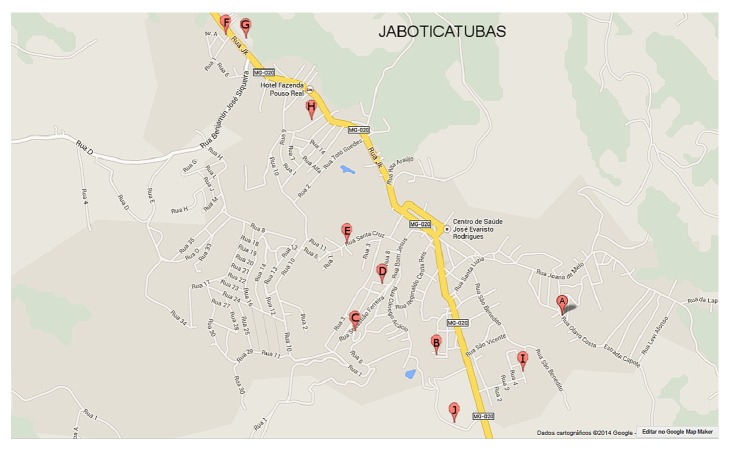
Spatial distribution of the entomological trapping sites in the urban area of Jaboticatubas (Minas Gerais state, Brazil). The study was conducted between May 2012 and April 2013.

The entomological captures that yielded data for the subsequent analyses were performed from May 2012 to April 2013, using HP light traps [10] placed in the peridomicile of ten houses in the urban area of Jaboticatubas. The trapping sites were labeled from A to J (Figure 1). The houses included in the study were selected based on the environmental conditions that favor the rearing of phlebotomine sand flies—such as shadowed areas, presence of domestic animals, and fruit trees—and previous reports of canine cases of leishmaniases in the neighborhood. The residents of the selected houses were informed of the project objectives and voluntarily signed a statement of informed consent prior to commencing the entomological captures. The trapping sites were georeferenced using a GARMIM-ETREX GPS.

The captures took place from 6:00 pm to 8:00 am, on three consecutive nights, always in the first week of each month. All captured male phlebotomine specimens were preserved in 70% ethanol and taken to the laboratory for species identification, while the females were placed in microtubes containing 6% DMSO and stored at −20°C until being required. For species identification, the head and the last three abdominal segments of every female were removed and slide-mounted with Berlese liquid. The remaining body parts were pooled and used for DNA extraction. The males were also slide-mounted with Berlese liquid.

We identified the phlebotomine sand flies of both genders using specific descriptions and taxonomic keys, as described by Young and Duncan [11]. Specimens that could not be identified due to missing or incomplete characters were considered* Lutzomyia* spp.* Lu. sallesi* and* Lu. cortelezzii* were considered* cortelezzii* complex, owing to the morphological similarity of their females [12]. Due to their high morphological resemblance, taxonomic identification of* Brumptomyia* females stopped at the genus level.

### 2.3. Climate Data

The monthly average maximum temperature (°C), total rainfall (mm), and relative humidity (%) data were sourced from the nearest meteorological station (fifth district of the Brazilian Institute of Meteorology, Belo Horizonte, MG). We employed the Spearman correlation analysis to evaluate the influence of climate variables on the population density of phlebotomine sand flies, using the Prism 6 software (GraphPad Inc., USA) with a 5% significance level. The results were expressed as the simple Spearman correlation coefficient (rs) for each pair of variables.

### 2.4. DNA Extraction from Phlebotomine Sand Flies

We extracted the total DNA from phlebotomine sand fly females using the Cell and Tissue Genomic Prep kit (GE Healthcare), after combining in a single pooled sample up to ten specimens of the same species, captured in the same month at the same capture site. Each DNA sample was identified by a number followed by the* Lutzomyia* species it was extracted from. The reliability of the DNA extraction was ensured by the amplification with genus-specific primers for* Lutzomyia* (5Llcac 5′ GTG GCC GAA CAT AAT GTT AG 3′ and 3Llcac 5′ CCA CGA ACA AGT TCA ACA TC 3′) in the cacophony IVS6 region, as described by other authors [13].

### 2.5. Nested PCR for* Leishmania* (*Ln*PCR)

The test for the presence of* Leishmania* DNA in the phlebotomine sand flies was carried out by* Leishmania* nested PCR (*Ln*PCR), specifically targeting the SSUrRNA gene [14–16]. Briefly, total DNA extracted from phlebotomine sand flies was first amplified with specific primers for the order Kinetoplastida but not exclusively for* Leishmania.* The resulting product of 603 bp was used as a template in the second PCR amplification in the presence of* Leishmania*-specific primers. Positive samples for* Leishmania* DNA showed a 353 bp fragment that was visualized under UV light after electrophoresis on 2% agarose gel and ethidium bromide staining. Negative (no DNA) and positive—DNA extracted from* Le. infantum* (MHOM/BR74/PP75)—controls were run in parallel.

### 2.6. *Leishmania* Species Identification and Phylogenetic Tree

The fragments amplified by* Ln*PCR were purified from agarose gels using a commercial kit (QIAquick Gel Extraction Kit, QIAGEN) and submitted to DNA sequencing, in both directions, using an appropriate kit (BigDye Terminator v3.1 Cycle) and the Megabace analyzer (GE HealthCare). Sequence editions and alignments against* Leishmania* DNA sequences [*Le. braziliensis* (M80292.1),* Le. amazonensis* (M80293.1), and* Le. chagasi* (M81430.1)] deposited in the GenBank database were performed using BioEdit tools (http://www.mbio.ncsu.edu/bioedit/bioedit.html) and the MacVector v. 11.0.2 software (MacVector Inc., Informax Inc., USA).

A consensus phylogenetic tree was constructed for* Leishmania* in the infected* Lutzomyia* phlebotomine sand flies using the distance-based unweighted pair group method with arithmetic mean (UPMGA). The analysis was performed with default parameters [bootstrap of 1,000 replicates, systematic tie breaking, Kimura 2-parameter, gamma correction off, estimate transversion ratio (Av. = 0.000), and proportionally distributed gaps] of the MacVector v. 11.0.2 software (MacVector Inc., Informax Inc., USA).

### 2.7. Minimum Infection Rates by* Leishmania* in the Phlebotomine Sand Flies

The minimum infection rates (MIR) by* Leishmania* in the captured phlebotomine sand flies were calculated as the ratio of the number of positive pools of each sand fly species and the number of specimens in that pool, multiplied by 100 [17].

## 3. Results

### 3.1. Phlebotomine Sand Fly Survey

**Figure 2:**
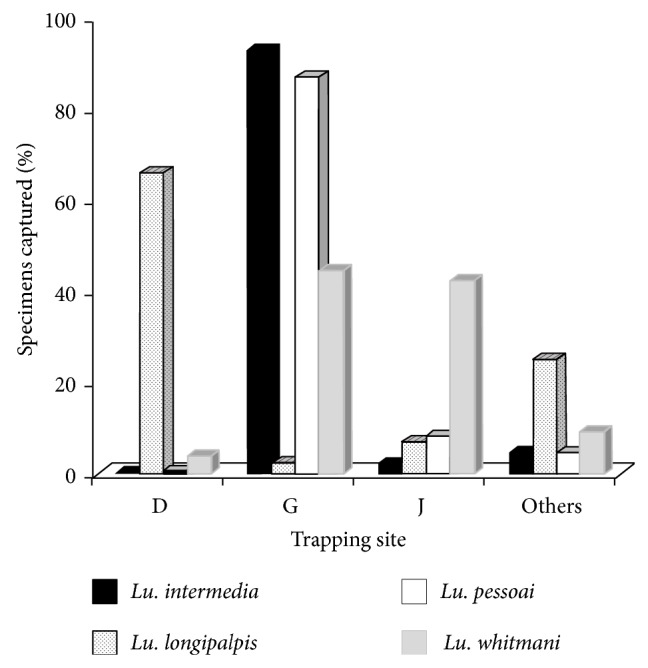
Distribution of vector species of leishmaniases according to entomological trapping sites in Jaboticatubas (Minas Gerais state, Brazil), between May 2012 and April 2013.

During the 12-month period included in the study, we captured 3,104 phlebotomine sand flies belonging to two genera,* Brumptomyia* and* Lutzomyia* (Table 1), with the overall male/female ratio of 2.3. Among the sixteen different species belonging to* Lutzomyia* genus, six are incriminated vectors of leishmaniases, namely,* Lu. fischeri*,* Lu. intermedia*,* Lu. migonei*,* Lu. pessoai*, and* Lu. whitmani* (as vectors of ACL) and* Lu. longipalpis* (vector of AVL) (Table 1). Three of the ten entomological trapping sites—D, G, and J—comprised about 86% of the total number of phlebotomine sand flies captured (Table 2). In addition, at these sites, at least one incriminated vector of leishmaniases as dominant species was observed (Figure 2).

### 3.2. Seasonal Variation Is the Phlebotomine Sand Fly Population

A positive correlation was found between the phlebotomine population density and the climate variables with correlation coefficients (rs) of 0.6719 for rainfall, 0.8792 for temperature, and 0.2035 for humidity. The correlation was statistically significant only for rainfall and temperature with *P* values of 0.0194 and 0.0003, respectively.

**Figure 3:**
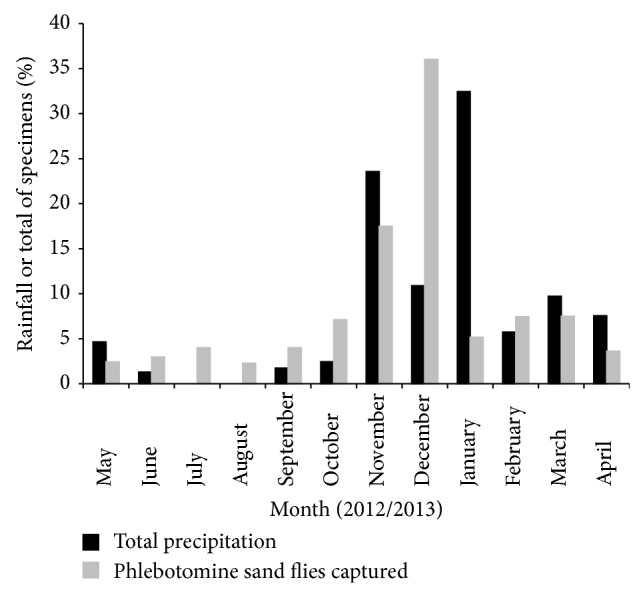
Influence of rainfall (black bars) on the population density of phlebotomine sand flies (grey bars) in the municipality of Jaboticatubas, Minas Gerais state, Brazil. The study was conducted between May 2012 and April 2013.

It is important to note the expressive increase in the population density during the short rainy season (November 2012−January 2013), accounting for 59% of the total number of specimens captured. As can be seen in Figure 3, a marked population increase occurred between two rainfall peaks.

### 3.3. Detection of* Leishmania* DNA in Phlebotomine Sand Flies

The DNA tests were performed on 249 species-specific pooled samples of* Lutzomyia* females, aiming to identify presence of the* Lutzomyia* cacophony gene. The expected 220 bp fragment was present in all samples (data not shown), confirming the reliability of the DNA extraction from the phlebotomine sand flies.

The 353 bp fragment characteristic of* Leishmania* genus was detected in 32 of the aforementioned 249 pooled samples of* Lutzomyia* (Table 3), with an overall MIR of 3.4%. Infected* Lutzomyia* specimens were captured from every trapping site (data not shown).

### 3.4. Specific Determination of the Infecting* Leishmania* in* Lutzomyia* Sand Flies

**Figure 4:**
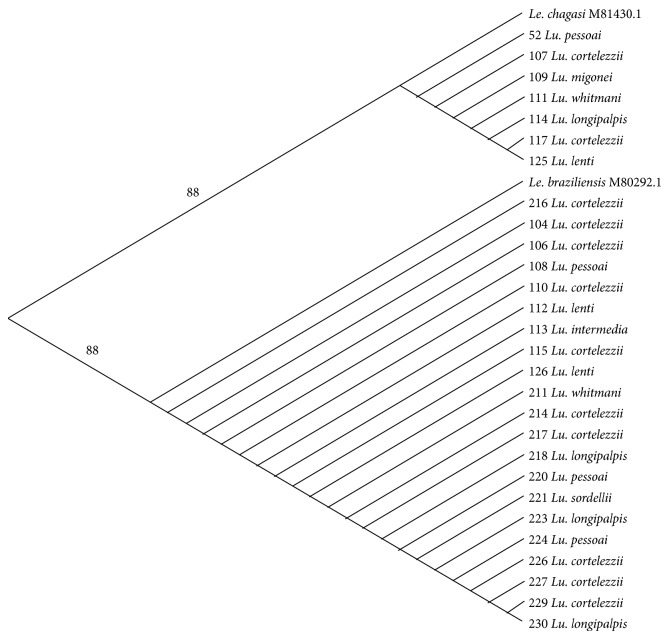
Phylogenetic tree (UPGMA) of* Leishmania* DNA identified in* Lutzomyia* phlebotomine sand flies captured in Jaboticatubas, state of Minas Gerais, Brazil. The bootstrap values are indicated on the branches. References:* Le. chagasi* M81430.1 and* Le. braziliensis* M80292.1. Test samples are identified by numbers followed by the* Lutzomyia* species carrying the* Leishmania* DNA. Study period: May 2012 to April 2013.

The nucleotide (nt) sequences of the 353 bp* Leishmania* fragments in the infected* Lutzomyia* phlebotomine sand flies were compatible with either* Le. infantum* or* Le. braziliensis. Le. amazonensis* was not detected in our samples. The alignment region containing the discriminating nucleotide mutations for the two* Leishmania* species is shown in the Supplementary Table available online at http://dx.doi.org/10.1155/2015/385493. A phylogenetic tree generated two clusters corresponding to* Le. infantum*- and* Le. braziliensis*-infected* Lutzomyia* samples (Figure 4). The infecting* Leishmania* species was successfully determined in 28 of the 32 samples of* Lutzomyia* (Table 3).

*Le. braziliensis,* an etiological agent of ACL, was detected in eight* Lutzomyia* species, namely,* Lu. intermedia, Lu. pessoai, Lu. whitmani, Lu. longipalpis, cortelezzii* complex,* Lu. lenti,* and* Lu. sordellii.* However, only the first three species are incriminated vectors of ACL, whereas* Lu. longipalpis* is vector of AVL. On the other hand, the causative agent of AVL—*Le. infantum*—was present not only in* Lu. longipalpis*, the proven vector of AVL, but also in the* cortelezzii* complex,* Lu. lenti*,* Lu. longipalpis, Lu. migonei, Lu. pessoai*, and* Lu. whitmani*.

## 4. Discussion

During the one-year study in Jaboticatubas, we captured an expressive number of* Lutzomyia* phlebotomine sand flies, belonging to sixteen different species. In accordance with the findings of several studies previously conducted in other Brazilian areas, the overall population density of* Lutzomyia* tended to increase significantly with rainfall and temperature, with most of the specimens captured during the short rainy season [18–28]. This profile may favor the planning of insect control actions.

Six incriminated vectors of leishmaniases were present, namely,* Lu. fischeri, Lu. intermedia, Lu. migonei, Lu. pessoai*, and* Lu. whitmani* (known vectors of ACL) and* Lu. longipalpis* (vector of AVL).* Lu. whitmani* was the most abundant species, accounting for 1/3 of the total number of the specimens captured in our study. This is a widely distributed species in both rural and urban areas and is amongst the most important vectors of ACL in Brazil [4, 29–35]. We found evidence that* Lu. whitmani* is separately infected by etiological vectors of both ACL and AVL, that is,* Le. braziliensis* and* Le. infantum*, respectively, in line with previous reports [4, 36, 37].

The second most numerous species in Jaboticatubas was* Lu. lenti*. This species has been commonly observed in savannah areas, where domestic animals, poultry in particular, are present in large numbers [38]. Although* Lu. lenti* appeared refractory to experimental* Leishmania* infection [39], natural infection lacking specific* Leishmania* identification has been reported by other authors [40]. We found* Lu. lenti* separately infected by* Le. infantum* and by* Le. braziliensis*.

*Lu. intermedia* is an important vector of ACL in the Southeast of Brazil [41–44] and, in this study, it was captured at a representative proportion (14.8%). The species is well adapted to various habitats, from forested to fully human-modified environments, particularly in areas where deforestation is gradually changing the epidemiological profile [4], as is the case in Jaboticatubas.

*Lu. pessoai,* another frequent species in our captures (8.1%), was suspected to be involved in ACL transmission in the 40s [45, 46]. Although this hypothesis was not subsequently confirmed, we found* Le. braziliensis* DNA in that species.

Specimens belonging to the* cortelezzii* complex, which are not amongst the incriminated leishmaniases vectors, were found to be separately infected by* Le. braziliensis* and* Le. infantum*. This is in accordance with previous reports in Brazil and Argentina [37, 47–49].

In addition to the most frequent* Lutzomyia* species discussed above, we also captured* Lu. evandroi, Lu. quinquefer, Lu. migonei*,* Lu. fischeri*, and* Lu. sordellii—*albeit in smaller numbers.* Lu. migonei* has been associated with ACL incidence in the Brazilian state of São Paulo [42] and some authors suggested that it could act as a secondary vector in the transmission of AVL, particularly in areas where* Lu. longipalpis* is absent [50–53]. Unfortunately, we were unable to determine the infecting* Leishmania* DNA, at specific level, in* Lu. migonei*.

*Lu. fischeri* is suspected as a secondary vector of ACL due to its high level of anthropophily and abundance in deforested areas, where sporadic cases of ACL have been reported [50]. However, there are no present or previous reports of natural* Leishmania* infection in that species.

In a previous study on spatial distribution of sand flies in the Brazilian state of Pernambuco,* Lu. sordellii* was found almost exclusively in forested areas [54]. In the past,* Lu. sordellii* was included in a group characterized by preferential feeding on cold-blooded animals, comprising* Lu. quinquefer*, among others [38]. Nevertheless,* Lu. quinquefer* was previously reported to be naturally infected by* Leishmania* sp. in Argentina [55] and, in the present study,* Lu. sordellii* infected by* Le. braziliensis* was identified.

*Lutzomyia* carrying* Leishmania* DNA were captured at every trapping site, indicating the wide distribution of the vector species throughout the urban area of Jaboticatubas. In three trapping sites (D, G, and J), the greatest population densities of* Lutzomyia* were recorded, with dominance of at least one leishmaniases vector.* Lu. intermedia* and* Lu. whitmani* were dominant in less-modified sites situated at the periphery of the urban area (G and J), whereas* Lu. longipalpis* was dominant at the center of Jaboticatubas, which is more urbanized (trapping site D). Although* Lu. longipalpis* was captured at much lower percentages than previously reported for other cities (72–92%) [22, 27, 28], the species dominance presently observed is compatible with the known adaptation of* Lu. longipalpis* to peridomiciles in human-modified areas. In addition to* Le. infantum* (AVL agent), we also captured* Lu. longipalpis* that was separately infected by* Le. braziliensis* (ACL agent).

Phylogenetic analysis revealed the clustering of the two circulating* Leishmania*, independently of the carrying* Lutzomyia* species. The finding of incriminated or suspected vectors of ACL with DNA of the etiological agent of AVL, and vice versa, suggests permissivity of some phlebotomine sand fly species to the infection by different* Leishmania* species. Furthermore,* Leishmania* DNA was identified in unexpected* Lutzomyia* species. Clearly, further studies are required to elucidate the vectorial competence of unsuspected phlebotomine sand flies species as well as the cross-infection of ACL/AVL vectors and parasites.* Leishmania* DNA findings do not imply that the infected* Lutzomyia* might play a role in leishmaniases transmission. According to Killick-Kendrick [56], four criteria must be fulfilled before incriminating any given species as vector of a zoonotic disease: (1) feeding on humans and on the animal reservoir, (2) providing support for the parasites after ingestion and expulsion of the infected blood meal, (3) displaying parasites indistinguishable from those isolated from patients, and (4) having the ability to transmit the parasite by biting.

The overall MIR in Jaboticatubas is comparable to those reported for endemic areas of leishmaniases [57, 58]. Moreover, the abundance and diversity of* Lutzomyia* species associated with the presence of infection by* Le. braziliensis* or* Le. infantum* indicate favorable conditions for the spreading of both ACL and AVL in an important tourism hub of Brazil. The presence of* Leishmania* DNA in unexpected* Lutzomyia* species and the cross-infection of etiological agent and vectors of AVL and ACL are a public health concern that deserves immediate attention. Further studies are needed on phlebotomine sand flies and* Leishmania* infection for a better understanding of the transmission cycle of leishmaniases in Jaboticatubas. Nevertheless, based on the population density of* Lutzomyia* and on the proved presence of etiological agents of leishmaniases, this tourism hub deserves special consideration, as timely and effective action may prevent spreading of these diseases to an even greater number of individuals.

## Supplementary Material

Multiple nucleotide alignment of *Leishmania* DNA carried by *Lutzomyia* captured in Jaboticatubas, state of Minas Gerais, Brazil. The discriminating mutations for the *Leishmania species* (at positions 38, 59 and 62) are marked with black arrows. Reference strains: *Le. chagasi* M81430.1 and *Le. braziliensis* M80292.1. Test samples are identified by numbers followed by the *Lutzomyia* species carrying the *Leishmania* DNA. Study period: May 2012−April 2013.

## Figures and Tables

**Table 1:** Phlebotomine sand flies captured in the municipality of Jaboticatubas, in the Brazilian state of Minas Gerais, during the May 2012–April 2013 study period, using HP-like light traps. The proven or incriminated vectors of leishmaniases are marked with ^*^.

Species		Number of specimens			Relative percentage
		Males	Females	Both genders	
*Brumptomyia* sp.		0	2	2	0.1
*cortelezzii *complex		76	105	181	5.8
*Lutzomyia aragaoi *		1	1	2	0.1
*Lu.evandroi *		0	1	1	0.0
*Lu. fischeri* ^*^		1	1	2	0.1
*Lu. intermedia* ^*^		376	77	453	14.6
*Lu. lenti *		482	260	742	23.9
*Lu. lloydi *		0	1	1	0.0
*Lu. longipalpis* ^*^		197	50	247	8.0
*Lu. lutziana *		2	4	6	0.2
*Lu. migonei* ^*^		31	5	36	1.2
*Lu. pessoai* ^*^		194	76	270	8.7
*Lu. quinquefer *		1	0	1	0.0
*Lu. renei *		0	2	2	0.1
*Lu. sordellii *		4	28	32	1.0
*Lu. termitophila *		1	7	8	0.3
*Lu. whitmani* ^*^		785	264	1,049	33.8
*Lutzomyia* spp.		12	57	69	2.2

Total	Number	2,163	941	3,104	—
	%	69.7	30.3	—	100.0

**Table 2:** Number of phlebotomine sand flies captured monthly, using HP light traps, in Jaboticatubas, in the Brazilian state of Minas Gerais, during the May 2012–April 2013 study period.

Year	Month	Number of specimens per trapping site					
		D	G	J	Others	Total	%
2012	May	25	13	31	6	75	2.4
	June	5	51	21	15	92	3.0
	July	12	55	44	13	124	4.0
	August	21	18	16	16	71	2.3
	September	20	87	5	12	124	4.0
	October	11	148	22	40	221	7.1
	November	70	355	30	88	543	17.5
	December	238	256	529	95	1,118	36.0

2013	January	7	61	51	41	160	5.2
	February	6	0	196	29	231	7.4
	March	3	115	69	46	233	7.5
	April	11	21	39	41	112	3.6

Total		429	1,180	1,053	442	3,104	100.0

**Table 3:** *Lutzomyia* species carrying *Leishmania* DNA captured in Jaboticatubas (MG, Brazil). Suspected or incriminated vectors of leishmaniases are marked with ^*^. Infecting *Leishmania* species were determined by nested PCR (*Ln*PCR). *Le.infantum* and *Le.braziliensis* are etiological agents of the American visceral (AVL) and of the American cutaneous (ACL) leishmaniases, respectively. Study period: May 2012–April 2013.

*Lutzomyia* species	*Leishmania* DNA identification			
	*Le*. *infantum *	*Le*. *braziliensis *	*Leishmania* sp.	Total
*cortelezzii* complex	1	8	2	11
*Lu. intermedia* ^*^	0	2	0	2
*Lu. lenti *	1	2	1	4
*Lu. longipalpis* ^*^	1	4	1	6
*Lu. migonei* ^*^	0	0	1	1
*Lu. pessoai* ^*^	0	3	1	4
*Lu. sordellii *	0	1	0	1
*Lu. whitmani* ^*^	1	1	1	3

Total	4	21	7	32
