# Supplementary material for: Phlebotomine Sand Fly Fauna and Leishmania Infection in the Vicinity of the Serra do Cipó National Park, a Natural Brazilian Heritage Site
Source: Biomed Res Int. 2015 Feb 22;2015:385493. doi: 10.1155/2015/385493 (PMC4352511; doi:10.1155/2015/385493)
Supplement: Supplementary file 1 — Multiple nucleotide alignment of Leishmania DNA carried by Lutzomyia captured in Jaboticatubas, state of Minas Gerais, Brazil. The discriminating mutations for the Leishmania species (at positions 38, 59 and 62) are marked with black arrows. Reference strains: Le. chagasi M81430.1 and Le. braziliensis M80292.1. Test samples are identified by numbers followed by the Lutzomyia species carrying the Leishmania DNA. Study period: May 2012−April 2013. [file 385493.f1.pdf]

Supplementary Table. Multiple nucleotide alignment of *Leishmania* DNA carried by *Lutzomyia* captured in Jaboticatubas, state of Minas Gerais, Brazil. The discriminating mutations for the *Leishmania* species (at positions 38, 59 and 62) are marked with black arrows. Reference strains: *Le. chagasi* M81430.1 and *Le. braziliensis* M80292.1. Test samples are identified by numbers followed by the *Lutzomyia* species carrying the *Leishmania* DNA. Study period: May 2012−April 2013.

	10 20 30
Le. chagasi M81430.1	T C C C A T C G C A A C C T C G G T T C G G T G T G T G G C G C C T T T
52 Lu. pessoai	T C C C A T C G C A A C C T C G G T T C G G T G T G T G G C G C C T T T
107 Lu. cortelezzii	T C C C A T C G C A A C C T C G G T T C G G T G T G T G G C G C C T T T
109 Lu. migonei	T C C C A T C G C A A C C T C G G T T C G G T G T G T G G C G C C T T T
111 Lu. whitmani	T C C C A T C G C A A C C T C G G T T C G G T G T G T G G C G C C T T T
114 Lu. longipalpis	T C C C A T C G C A A C C T C G G T T C G G T G T G T G G C G C C T T T
117 Lu. cortelezzii	T C C C A T C G C A A C C T C G G T T C G G T G T G T G G C G C C T T T
125 Lu. lenti	T C C C A T C G C A A C C T C G G T T C G G T G T G T G G C G C C T T T
Le. braziliensis M80292.1	T C C C A T C G C A A C t T C G G T T C G G T G T G T G G C G C C T T T
216 Lu. cortelezzii	T C C C A T C G C A A C C T C G G T T C G G T G T G T G G C G C C T T T
104 Lu. cortelezzii	T C C C A T C G C A A C C T C G G T T C G G T G T G T G G C G C C T T T
106 Lu. cortelezzii	T C C C A T C G C A A C C T C G G T T C G G T G T G T G G C G C C T T T
108 Lu. pessoai	T C C C A T C G C A A C C T C G G T T C G G T G T G T G G C G C C T T T
110 Lu. cortelezzii	T C C C A T C G C A A C C T C G G T T C G G T G T G T G G C G C C T T T
112 Lu. lenti	T C C C A T C G C A A C C T C G G T T C G G T G T G T G G C G C C T T T
113 Lu. intermedia	T C C C A T C G C A A C C T C G G T T C G G T G T G T G G C G C C T T T
115 Lu. cortelezzii	T C C C A T C G C A A C C T C G G T T C G G T G T G T G G C G C C T T T
126 Lu. lenti	T C C C A T C G C A A C C T C G G T T C G G T G T G T G G C G C C T T T
211 Lu. whitmani	T C C C A T C G C A A C C T C G G T T C G G T G T G T G G C G C C T T T
214 Lu. cortelezzii	T C C C A T C G C A A C C T C G G T T C G G T G T G T G G C G C C T T T
217 Lu. cortelezzii	T C C C A T C G C A A C C T C G G T T C G G T G T G T G G C G C C T T T
218 Lu. longipalpis	T C C C A T C G C A A C C T C G G T T C G G T G T G T G G C G C C T T T
220 Lu. pessoai	T C C C A T C G C A A C C T C G G T T C G G T G T G T G G C G C C T T T
221 Lu. sordelli	T C C C A T C G C A A C C T C G G T T C G G T G T G T G G C G C C T T T
223 Lu. longipalpis	T C C C A T C G C A A C C T C G G T T C G G T G T G T G G C G C C T T T
224 Lu. pessoai	T C C C A T C G C A A C C T C G G T T C G G T G T G T G G C G C C T T T
226 Lu. cortelezzii	T C C C A T C G C A A C C T C G G T T C G G T G T G T G G C G C C T T T
227 Lu. cortelezzii	T C C C A T C G C A A C C T C G G T T C G G T G T G T G G C G C C T T T
229 Lu. cortelezzii	T C C C A T C G C A A C C T C G G T T C G G T G T G T G G C G C C T T T
230 Lu. longipalpis	T C C C A T C G C A A C C T C G G T T C G G T G T G T G G C G C C T T T

	40 50 60 70
Le. chagasi M81430.1	G - A G G G G T T T A G T G C G T C C G G T a C G a G C T C C G G T T C
52 Lu. pessoai	G - A G G G G T T T A G T G C G T C C G G T a C G a G C T C C G G T T C
107 Lu. cortelezzii	G - A G G G G T T T A G T G C G T C C G G T a C G a G C T C C G G T T C
109 Lu. migonei	G - A G G G G T T T A G T G C G T C C G G T a C G a G C T C C G G T T C
111 Lu. whitmani	G - A G G G G T T T A G T G C G T C C G G T a C G a G C T C C G G T T C
114 Lu. longipalpis	G - A G G G G T T T A G T G C G T C C G G T a C G a G C T C C G G T T C
117 Lu. cortelezzii	G - A G G G G T T T A G T G C G T C C G G T a C G a G C T C C G G T T C
125 Lu. lenti	G - A G G G G T T T A G T G C G T C C G G T a C G a G C T C C G G T T C
Le. braziliensis M80292.1	G G A G G G G T T T A G T G C G T C C G G T G C G G G C T C C G G T T C
216 Lu. cortelezzii	G G A G G G G T T c A G T G C G T C C G G T G C G G G C T C C G G T T C
104 Lu. cortelezzii	G G A G G G G T T T A G T G C G T C C G G T G C G G G C T C C G G T T C
106 Lu. cortelezzii	G G A G G G G T T T A G T G C G T C C G G T G C G G G C T C C G G T T C
108 Lu. pessoai	G G A G G G G T T T A G T G C G T C C G G T G C G G G C T C C G G T T C
110 Lu. cortelezzii	G G A G G G G T T T A G T G C G T C C G G T G C G G G C T C C G G T T C
112 Lu. lenti	G G A G G G G T T T A G T G C G T C C G G T G C G G G C T C C G G T T C
113 Lu. intermedia	G G A G G G G T T T A G T G C G T C C G G T G C G G G C T C C G G T T C
115 Lu. cortelezzii	G G A G G G G T T T A G T G C G T C C G G T G C G G G C T C C G G T T C
126 Lu. lenti	G G A G G G G T T T A G T G C G T C C G G T G C G G G C T C C G G T T C
211 Lu. whitmani	G G A G G G G T T T A G T G C G T C C G G T G C G G G C T C C G G T T C
214 Lu. cortelezzii	G G A G G G G T T T A G T G C G T C C G G T G C G G G C T C C G G T T C
217 Lu. cortelezzii	G G A G G G G T T T A G T G C G T C C G G T G C G G G C T C C G G T T C
218 Lu. longipalpis	G G A G G G G T T T A G T G C G T C C G G T G C G G G C T C C G G T T C
220 Lu. pessoai	G G A G G G G T T T A G T G C G T C C G G T G C G G G C T C C G G T T C
221 Lu. sordelli	G G A G G G G T T T A G T G C G T C C G G T G C G G G C T C C G G T T C
223 Lu. longipalpis	G G A G G G G T T T A G T G C G T C C G G T G C G G G C T C C G G T T C
224 Lu. pessoai	G G A G G G G T T T A G T G C G T C C G G T G C G G G C T C C G G T T C
226 Lu. cortelezzii	G G A G G G G T T T A G T G C G T C C G G T G C G G G C T C C G G T T C
227 Lu. cortelezzii	G G A G G G G T T T A G T G C G T C C G G T G C G G G C T C C G G T T C
229 Lu. cortelezzii	G G A G G G G T T T A G T G C G T C C G G T G C G G G C T C C G G T T C
230 Lu. longipalpis	G G A G G G G T T T A G T G C G T C C G G T G C G G G C T C C G G T T C
